# Impact of the COVID-19 Pandemic on Routine Childhood Immunization in Saudi Arabia

**DOI:** 10.3390/vaccines8040581

**Published:** 2020-10-03

**Authors:** Mohammed Alsuhaibani, Aqeel Alaqeel

**Affiliations:** Department of Pediatrics, College of Medicine, Qassim University, Qassim 51452, Saudi Arabia; a.alaqeel@qumed.edu.sa

**Keywords:** childhood, children, immunizations, vaccinations, COVID-19, coronavirus, vaccine-preventable disease, SARS-CoV-2

## Abstract

The COVID-19 pandemic is impacting national and international public health. Routine childhood immunization may be adversely affected by COVID-19 mitigation measures. We aimed to identify the prevalence of delayed immunization and explore the reasons and barriers for delayed immunization during the COVID-19 pandemic in the Qassim region, Saudi Arabia. We conducted a cross-sectional study using an online self-administered questionnaire for parents of children under two years of age during the period from 1 May to 30 June 2020. Most of the 749 participants (82.6%) were mothers, with 31 to 40 years being the most common age group (49.8%). Nearly three-quarters (73.2%) of the parents had appointments scheduled for their child’s vaccination during the pandemic, and approximately 23.4% of the parents reported a delay of more than one month in the immunization of their child. The most common reason for the delay was the fear of being infected by COVID-19 (60.9%). Large household size and lack of insurance were risk factors for immunization delay. The COVID-19 pandemic has affected the timeliness of routine childhood immunization in Saudi Arabia. Childhood immunization should be prioritized, as well as the implementation of focused strategies to achieve significant and sustainable vaccination rates during pandemics.

## 1. Introduction

The coronavirus disease 2019 (COVID-19) pandemic is a threat to public health and healthcare systems worldwide [1]; prompting governments to implement numerous interventions such as curfews, extensive screening measures and travel bans to prevent the further spread of COVID-19 [2]. School closure has been a vital intervention strategy to curb community transmission during the pandemic [3,4]. Although these strategies are crucial, concerns have arisen regarding lengthy school closures and home quarantine, which may negatively impact children’s mental and physical health [2,5]. Furthermore, disruptions to healthcare services may result in childhood immunization being missed or delayed, which is a significant concern [6,7]. Vaccine-preventable disease (VPD) remains an important issue that requires adherence to recommendations to prevent severe illness in children [8]. Delayed or missed vaccination makes children susceptible to certain preventable diseases and may also affect herd immunity [9]. According to the World Health Organization (WHO), VPD is a threat to 80 million children worldwide because of the disruption of healthcare systems due to the COVID-19 pandemic [10]. In addition, vaccination against influenza may help decrease the severity of disease in patients who experience co-morbid COVID-19 and influenza infections [11,12].

Prior to the COVID-19 pandemic, the percentage of deferred vaccinations varied across countries. For instance, only 25% of children received vaccinations timeously in the United States [12,13]. In Saudi Arabia, the delay of vaccinations ranged from 9% to 24% due to various factors, such as unavailability of the vaccine in some primary healthcare facilities and traveling when vaccination was due [14,15].

Administering vaccinations in a timely manner is crucial but may be affected by various factors. Natural disasters, for example, may have a negative impact on vaccination rates and timeliness, leading to infectious disease outbreaks [6,16]. Thus, the American Academy of Pediatrics recommends avoiding the disruption of childhood immunization to prevent VPD outbreaks [17]. The Saudi Ministry of Health (MOH) also recommends that childhood vaccinations should be given timeously during the COVID-19 pandemic.

To date, there are few reports regarding the impact of the COVID-19 pandemic on childhood immunization. In this study, we aimed to identify the prevalence of delayed vaccinations and uncover the reasons and barriers for delayed immunization during the COVID-19 pandemic. We hypothesized that the COVID-19 pandemic may exacerbate delays in childhood vaccinations. The findings of our research will be pertinent to recognizing the obstacles to vaccination timeliness during public health emergencies and thereby aid in the implementation of strategies at the national level for pandemic preparedness.

## 2. Materials and Methods

### 2.1. Population and Study Design

A descriptive, cross-sectional study was conducted using an online self-administered questionnaire comprising 25 questions. The questionnaire was designed to measure the prevalence of, and reasons for, delays in childhood vaccinations during the COVID-19 pandemic in Saudi Arabia. The study population included parents from different cities of the Qassim region who had a child under two years of age during the period from 1 March to 30 June 2020. Parents who did not have children below the age of two were excluded. We conducted a nonprobability convenience sampling of parents via the WhatsApp application using the Google platform between 1 May and 30 June. We chose 12 medical students to distribute the questionnaire and collect data from parents in different cities in Qassim. The participants gave consent before commencing the questionnaire.

### 2.2. Study Context

The Qassim region is located in the center of Saudi Arabia and has a population of approximately 1.5 million, of which 80.4% are Saudi citizens. The national childhood immunization schedule targets 16 VPDs at various stages, beginning at birth and subsequently at 2, 4, 6, 9, 12, 18 and 24 months. The vaccines are for hepatitis B, diphtheria–tetanus–pertussis, polio, *Hemophilus influenzae* type B, rotavirus, pneumococcal infections, bacillus Calmette–Guérin (BCG), hepatitis A, measles, varicella, meningococcal infections, measles-mumps-rubella (MMR) and human papillomavirus (HPV).

### 2.3. Questionnaire

The questionnaire was validated in two steps. Initially, it was reviewed by three academic experts in the field to evaluate the quality and bias in the survey. Then, the questionnaire was pretested on 20 parents who were subsequently excluded from the current analysis. Minor language modification was performed based on expert suggestions. The survey was conducted in Arabic for the participants.

The questionnaire comprised four sections: parents’ demographic data; current immunization status and attitude toward immunization delay; immunization during the COVID-19 pandemic; and possible reasons that prevented parents from vaccinating their child or children.

Vaccination delay was defined as vaccinations that took place one month or more after the designated time.

### 2.4. Data Analysis

Parents’ attitudes were recorded on a 5-point Likert scale ranging from “strongly disagree” to “strongly agree”. Responses such as “agree” and “strongly agree” were coded with 1 point. Responses such as “neutral,” “disagree,” and “strongly disagree” were coded with 0 points. A negative question was included and reverse-coded to avoid bias. The overall score of attitudes was calculated by summing the scores of the 3 questions. The attitude score range was from 0 to 3 points, which indicated that the higher the score, the more positive the attitude toward the timeliness of vaccinations during the COVID-19 pandemic. By using a score of 2 as the cutoff point to determine the level of attitude, parents were classified as having a negative attitude if the score was from 0 to 1 point, and as having a positive attitude if the score was from 2 to 3 points.

Data were summarized with numerical values, percentages, means and standard deviation whenever appropriate. For comparisons, a Mann–Whitney U test or a Kruskal–Wallis test was applied. Normality, statistical interactions and collinearity (i.e., variance inflation factor) were also assessed using Kolmogorov–Smirnov and Shapiro–Wilk tests. The relationship between length of delay in childhood vaccination, consistent, timely childhood vaccinations and preferred vaccination locale during the pandemic was calculated using a chi-squared test. A *p*-value of <0.05 was considered significant for all statistical tests. All data analyses were performed using IBM SPSS Statistics for Windows, version 21.0.

### 2.5. Ethical Considerations

Approval from the Qassim regional research ethics committee was granted (IRB# H-04-Q-001).

## 3. Results

In total, 802 parents were invited to participate in the study, and 53 participants were excluded due to incomplete questionnaires. We included 749 participants in the analysis, as shown in Table 1. Mothers were predominantly higher in number (82.6%) than fathers (17.4%), with 31 to 40 years being the most common age group (49.8%). Most parents obtained a bachelor’s degree (78.4%). Findings showed that 9.9% of the participants were working in the medical field. Furthermore, 38.9% reported having one child. Nearly all participants (93.5%) stated that they currently had one child under two years.

In assessing awareness of COVID-19 risk in children, our findings showed that the proportion of parents who believed “children could be infected with COVID-19”, “children can transmit COVID-19” and “COVID-19 illness in children can lead to child hospitalization” were 73.2%, 74.2% and 73.4%, respectively.

The parents’ attitudes regarding the timeliness of childhood vaccination showed that the proportion of parents who “strongly agreed” regarding the statement “vaccinations are essential to keep children healthy” and “vaccinations should be given at the scheduled time” were 73.6% and 53.9%, respectively. Conversely, the proportion of parents who “strongly agreed” regarding the statement “vaccination delay is not a problem as you give all vaccines regardless of the due time for your child” was low, with only 6.3%. Based on these three attitude statements, the mean attitude score was 2.25 (SD = 0.73), with nearly all parents (90%) classified as having a positive attitude, while the rest (10%) were classified as negative. When comparing the attitude score versus the sociodemographic characteristics of parents, it was observed that parents with one child were significantly more likely to have better attitudes than those with two or more children (F = 2.712; *p* = 0.006), as shown in Table 2.

Overall, 73.2% of the parents had appointments scheduled for their child’s vaccination during the COVID-19 pandemic. Furthermore, approximately 47.8% of the parents reported a delay of more than two weeks in the immunization of their child. A significant delay of more than one month was observed in 23.4% of the cases. However, 52.2% reported that the scheduled vaccine was given on time or within two weeks of the due date. Almost 73% of the respondents were aware of the MOH recommendation. The most common source of information was social media sites (49.5%).

**Figure 1:**
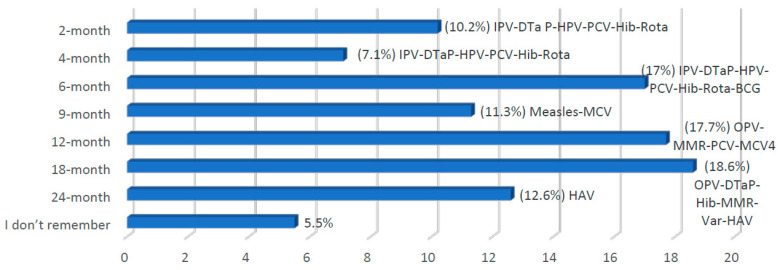
Frequency of vaccine delay by scheduled age. This figure shows the most frequently delayed vaccination ages. IPV—injectable polio vaccine; DTaP—diphtheria-tetanus-pertussis; HPV—Hepatitis B vaccine; PCV—pneumococcal conjugate vaccine; Hib—*Hemophilus influenzae* type B; Rota—rotavirus; BCG—bacillus Calmette–Guérin; MCV—meningococcal; OPV—oral polio; MMR—measles-mumps-rubella; Var—varicella; HAV—hepatitis A vaccine.

Of those who had a child scheduled for immunization during the pandemic, 73% were aware of MOH recommendations for vaccinations. Nearly 70% of the parents received this information through social media, and 30% through TV or newspapers. The most frequently missed vaccinations are shown in Figure 1.

**Figure 2:**
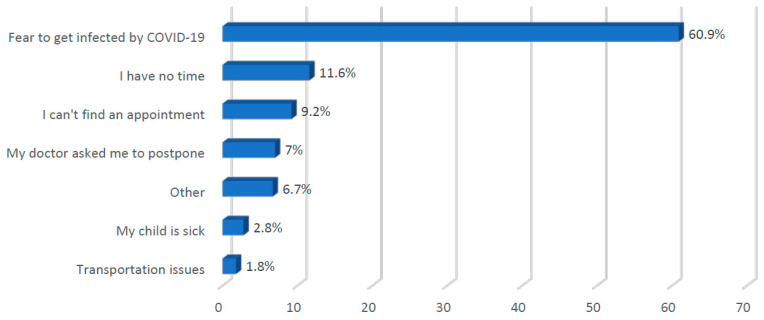
Reasons for vaccination delays.

Figure 2 shows the parents’ reasons for delaying vaccinations, indicating that the most common reason was fear of being infected with COVID-19 (60.9%), followed by time constraints (11.6%) and difficulties in scheduling an appointment (9.2%). Other reasons mentioned by parents were traveling during the vaccination time, vaccine unavailability or closed clinics (6.7%).

**Figure 3:**
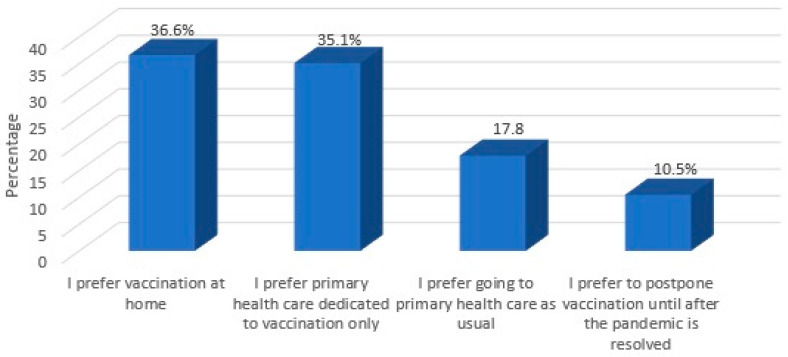
Preferred locale for child vaccination during the pandemic.

When parents were asked about their preferred locale for vaccination, most of the parents preferred to vaccinate their children at home (36.6%) or at primary healthcare facilities dedicated to vaccination only (35.1%). However, only 17.8% reported that they would continue to obtain childhood vaccinations at hospitals or other healthcare centers as usual (see Figure 3).

Table 3 depicts the univariate and multivariate regression analyses to determine which factors were associated with delayed vaccination of more than one month among parents who had scheduled child vaccinations during the pandemic. Findings revealed that the chance of delaying vaccinations for participants in the 31–40 years age group was twice as high as that of the 18–30 years age group (*p* = 0.011); however, in the multivariate regression model, the results became insignificant. The univariate and multivariate regression analyses showed that the chance of delayed vaccination for participants with multiple children was three times more likely than for those with only one child (*p* < 0.001). Additionally, participants with medical insurance were less likely to delay their children’s vaccinations than those without medical insurance (*p* = 0.043).

## 4. Discussion

The present study assessed the prevalence of, and reasons for, delayed childhood vaccinations during the COVID-19 pandemic. Our results indicate that the COVID-19 pandemic affected the timeliness of childhood immunization in the Qassim region of Saudi Arabia. To our knowledge, this is the first study to measure the effects of the COVID-19 pandemic on childhood immunization in Saudi Arabia.

Several reports have suggested that childhood vaccination has been affected by the pandemic. Abbas et al. suggested that the risk of death due to VPD outweighed the risk of death due to possibly contracting COVID-19 during clinic visits [18]. After the national emergency declaration in the USA, a reduction in routine vaccinations was observed, primarily in children older than one month [19]. Moreover, an almost 20% decline in MMR vaccination was observed in England. The hexavalent vaccination also decreased, although less significantly than that of MMR [20]. However, in Pakistan, a substantial reduction in immunization of 52% was reported during the lockdown period. Reports indicate that the outreach vaccination service was affected more than fixed center services [21]. In our study, parents reported an almost 24% vaccination delay in routine vaccination during the first three months of the pandemic. The results of previous reports are consistent with our findings. However, previous reports were dependent on vaccination prescriptions to measure the trend of vaccination. In our study, we relied on parental reports regarding vaccination delays.

In the current study, household size was a risk factor for vaccination delay, consistent with previous research confirming the association between larger household size and vaccination delay among children [15,22]. This may be due to an increase in parental tasks, causing parents to miss vaccinations as a result of being overwhelmed. Moreover, uninsured families were at risk of delayed vaccination. In Saudi Arabia, vaccination is provided free of charge for all families under the government’s primary healthcare system. However, insured families also have access to care from the private sector as well as the government’s primary healthcare system. On the other hand, two-thirds of the delayed vaccinations occurred among children aged 12 months and younger. This finding is inconsistent with a report from the USA, in which they targeted and promoted vaccination in younger age groups during the pandemic. Indeed, routine childhood immunization should be prioritized in younger children.

The study revealed that most parents had a positive attitude toward the importance of timely vaccinations. In contrast, a previous study in Saudi Arabia showed that 65% of parents were not concerned about vaccination delays. The difference in findings may be attributed to improved parental vaccination awareness through primary care providers, MOH and the media [14]. Prior international studies have found parental education to be significantly associated with a positive attitude toward different types of vaccinations [23,24].

As expected, the fear of contracting COVID-19 was the most common reason for vaccination delays. Further reasons included the unavailability of vaccination appointments due to the lockdown and resultant healthcare service limitations. This is consistent with findings indicating the potential causes of missed vaccinations during the pandemic in the USA [19]. Surprisingly, advice received from healthcare service providers was another reason given by the parents to delay vaccinations. This may be due to the ambiguity of immunization recommendations and initial stay-at-home orders at the onset of the pandemic. Due to the fear of COVID-19 infection, most families who delayed their child’s vaccination preferred to get their child vaccinated at home or attended to by healthcare staff solely dedicated to administering vaccinations. In Saudi Arabia, there was a home vaccination program during the influenza season aimed at increasing influenza vaccine coverage [25]. Thus, implementing home vaccination during a pandemic should be considered to increase vaccination rates, particularly for high-risk children. Moreover, many healthcare service providers in the USA have allotted vaccination appointments exclusively to the mornings, while sick patients are scheduled for later in the day. Other practices request that families wait in their vehicles until the exam room is ready, thereby avoiding contact between families [19,26].

While the findings of this study are significant, the study is not without limitations. Specifically, the study was conducted in a single region in Saudi Arabia that may not be representative of the entire population. Additionally, the measurement of attitude may be imprecise due to the limited number of questions. Moreover, this study may have introduced some recall bias, and the results may be influenced by subjectivity. Nevertheless, this study provides an important understanding of the overall picture regarding immunization delay in Saudi Arabia during the pandemic. Thus, the findings may be used as a reference for outlining strategies to improve routine vaccination program coverage during natural disasters and pandemics.

## 5. Conclusions

We identified that the COVID-19 pandemic affected the timeliness of routine childhood vaccinations in Saudi Arabia. The results of our study demonstrated numerous barriers to timeous immunization. We suggest that routine childhood vaccination be prioritized and focused strategies implemented to achieve a significant and sustainable increase in vaccinations during pandemics.

## Figures and Tables

**Table 1:** Sociodemographic characteristics of parents (*n* = 749).

Study Variables	N (%)
Parent’s gender	
Father	130 (17.4%)
Mother	619 (82.6%)
Age group	
18–30 years	274 (36.6%)
31–40 years	373 (49.8%)
41–50 years	91 (12.1%)
51–60 years	08 (12.1%)
>60 years	03 (0.40%)
Educational level	
Secondary or less	110 (14.7%)
Bachelor’s degree	587 (78.4%)
Master’s or PhD	52 (06.9%)
Monthly income in SAR	
Not mentioned	136 (18.2%)
<2000	62 (08.3%)
2000–6000	135 (18.0%)
6001–10,000	155 (20.7%)
10,001–15,000	160 (21.4%)
>15,000	101 (13.5%)
Working in medical field	
Yes	74 (09.9%)
No	675 (90.1%)
Number of children under 2 years old	
One	700 (93.5%)
Two	49 (06.5%)
Number of children	
One	291 (38.9%)
Two	193 (25.8%)
Three	111 (14.8%)
Four	84 (11.2%)
Five or more	70 (09.3%)
Having medical insurance	
Yes	149 (19.9%)
No	600 (80.1%)
Is your child up to date with the national vaccination program?	
Yes	687 (91.7%)
No	19 (02.5%)
Not all vaccines	32 (04.3%)
I do not know	11 (01.5%)
Where does your child receive his/her vaccinations?	
Government hospital/PHC	630 (84.1%)
Private hospital	42 (05.6%)
Both	77 (10.3%)

SAR—Saudi riyal; PHC—primary healthcare center.

**Table 2:** Assessment of parents’ attitudes concerning childhood vaccination timeliness during COVID-19 (*n* = 749).

Attitude Statement	SA N (%)	A N (%)	N N (%)	D N (%)	SD N (%)
Vaccinations are essential to keep children healthy	551 (73.6%)	156 (20.8%)	36 (04.8%)	03 (0.40%)	03 (0.40%)
Vaccination should be given at the time	404 (53.9%)	277 (37.0%)	57 (07.6%)	09 (01.2%)	02 (0.30%)
Vaccination delay is not a problem as you give all vaccines regardless the due time for your child	47 (06.3%)	267 (35.6%)	137 (18.3%)	215 (28.7%)	83 (11.1%)

SA—strongly agree; A—agree; N; neutral; D—disagree; SD—strongly disagree.

**Table 3:** Univariate and multivariate regression model for determining factors associated with delayed vaccination (more than one month) (*n* = 548).

Factor	Delayed N	UOR (95% CI)	*p*-Value	AOR (95% CI)	*p*-Value
Parents gender					
Father	23/84	Ref		Ref	
Mother	105/464	0.78 (0.46–1.31)	0.344	0.73 (0.40–1.34)	0.310
Age group					
18–30 years	38/209	Ref		Ref	
31–40 years	67/269	2.20 (1.19–4.05)	0.011 **	1.12 (0.54–2.33)	0.767
>40 years	23/70	1.47 (0.83–2.61)	0.181	1.08 (0.58–2.03)	0.802
Educational level					
Secondary or less	17/60	Ref		Ref	
Bachelor’s degree or higher	111/488	0.74 (0.41–1.36)	0.336	0.76 (0.40–1.44)	0.399
Monthly income in SAR					
Not answered	18/98	Ref		Ref	
≤10,000	67/265	1.35 (0.73–2.49)	0.344	1.27 (0.65–2.47)	0.488
>10,000	43/185	0.89 (0.58–1.39)	0.620	0.86 (0.53–1.41)	0.550
Working in medical field					
Yes	7/49	Ref		Ref	
No	121/499	1.92 (0.84–4.39)	0.121	2.35 (0.96–5.74)	0.061
Number of children < 2 years					
One	115/511	Ref		Ref	
Two	13/37	1.86 (0.92–3.78)	0.084	1.61 (0.76–3.42)	0.218
Number of children					
One	33/212	Ref		Ref	
Two to three	53/228	3.45 (2.02–5.90)	<0.001 **	3.06 (1.65–5.66)	<0.001 **
Four or more	42/108	2.10 (1.28–3.44)	0.003 **	2.06 (1.19–3.55)	0.009 **
Having medical insurance					
Yes	16/116	0.46 (0.26–0.81)	0.007 **	0.54 (0.29–0.98)	0.043 **
No	112/432	Ref		Ref	

UOR—unadjusted odds ratio; AOR—adjusted odds ratio; CI—confidence interval; SAR—Saudi riyal. ** Significant at *p* < 0.05 level.
